# Dynamic Default Mode Network across Different Brain States

**DOI:** 10.1038/srep46088

**Published:** 2017-04-06

**Authors:** Pan Lin, Yong Yang, Junfeng Gao, Nicola De Pisapia, Sheng Ge, Xiang Wang, Chun S. Zuo, James Jonathan Levitt, Chen Niu

**Affiliations:** 1 College of Biomedical Engineering, South-Central University for Nationalities, Wuhan, 430074, China; 2 School of Information Technology, Jiangxi University of Finance and Economics, Nanchang, 330013, China; 3 Key Laboratory of Biomedical Information Engineering of Education Ministry, Institute of Biomedical Engineering, Xi’an Jiaotong University, Xi’an 710049, China; 4 Center for Mind/Brain Sciences, University of Trento, Mattarello, 38100, Italy; 5 Key Laboratory of Child Development and Leaning Science of Ministry of Education, Research Center for Learning Science, Southeast University, Nanjing, Jiangsu, 210096, China; 6 Medical Psychological Institute of Second Xiangya Hospital, Central South University, Changsha, 410011, China; 7 Brain Imaging Center, McLean Hospital, Department of Psychiatry, Harvard Medical School, Belmont, MA, 02478, USA; 8 Clinical Neuroscience Division, Laboratory of Neuroscience, Department of Psychiatry, VA, Boston Healthcare System, Brockton Division, and Harvard Medical School, Boston, MA 02301, USA; 9 Psychiatry Neuroimaging Laboratory, Department of Psychiatry, Brigham & Women’s Hospital, Harvard Medical School, Boston, MA 02215, USA; 10 Department of Medical Imaging, First Affiliated Hospital of Xi’an Jiaotong University College of Medicine, Shaanxi Xi’an 710061, China

## Abstract

The default mode network (DMN) is a complex dynamic network that is critical for understanding cognitive function. However, whether dynamic topological reconfiguration of the DMN occurs across different brain states, and whether this potential reorganization is associated with prior learning or experience is unclear. To better understand the temporally changing topology of the DMN, we investigated both nodal and global dynamic DMN-topology metrics across different brain states. We found that DMN topology changes over time and those different patterns are associated with different brain states. Further, the nodal and global topological organization can be rebuilt by different brain states. These results indicate that the post-task, resting-state topology of the brain network is dynamically altered as a function of immediately prior cognitive experience, and that these modulated networks are assembled in the subsequent state. Together, these findings suggest that the changing topology of the DMN may play an important role in characterizing brain states.

Recent studies have revealed higher metabolic activity in a particular network of regions termed the default mode network (DMN)^1^,^2^,^3^. The DMN is a set of functionally and structurally connected brain regions that typically exhibit deactivation during the performance of an externally oriented attention-demanding task and high cerebral blood flow and oxygen consumption during the resting state^4^,^5^,^6^,^7^. DMN function has been largely linked to self-referential thought, mind-wandering, internal-oriented cognition, and autobiographical memory^2^,^7^. To date, a large body of studies use a stationary functional connectivity (FC) approach to characterize DMN organization via analyses of inter-regional temporal relationships during resting and task states^8^,^9^,^10^,^11^,^12^,^13^. The various neuroimaging techniques used for stationary FC analysis have revealed DMN spatial-temporal properties not only during attention-demanding cognitive task and at rest, but also during anaesthesia, in vegetative patients, and during different stages of sleep^14^,^15^,^16^,^17^. Moreover, accumulating evidence indicates that stationary FC within the DMN is linked to specialized cognitive functions and clinically relevant information^8^,^12^,^18^,^19^,^20^,^21^.

The brain is a complex system in which the dynamic adjustment of network organization over multiple time scales is crucial for mediating perception and cognition^22^,^23^,^24^,^25^,^26^,^27^,^28^,^29^,^30^,^31^,^32^. Characterizing the dynamics of brain FC or properties of network topology is thought to be important for gaining a better understanding of brain function and behavioural performance^13^,^33^,^34^,^35^. Further, growing evidence supports the idea that dynamic FC patterns are tightly linked to adaptive behaviour^36^.

The flexibility of functional network configurations during learning tasks has been shown to facilitate subsequent learning performance^37^,^38^,^39^. Additional evidence suggests that brain functional networks dynamically adjust the structure of their global and local network connectivity as a means to guide optimal behavioural performance, such as during an upcoming visual discrimination task^40^,^41^. These findings emphasize the importance of dynamic coordination within the brain network and the adaptability of its topological structure for supporting ongoing task performance^42^,^43^.

Until now, few studies have directly examined dynamic DMN during different brain states (i.e., pre-task resting state, task state, and post-task resting state). Answering how DMN temporal properties depend on different brain states, such as past cognitive learning experiences, could thus help provide a more complete understanding of DMN function. Studies have found that prior tasks can affect post-task resting-state brain activity^44^,^45^,^46^. Thus, modulation of learning-dependent spontaneous brain activity has been observed after people perform cognitive tasks involving working memory, emotion, visual perception, and motor training^44^,^45^,^47^,^48^,^49^,^50^. Such modulation of brain regions associated with a prior task can be understood as reflecting the gradual establishment, or reinforcement, of recent experiences in the offline resting state. A possible explanation for this is that recent cognitive processes require a functional interaction between multiple specialized local and remote brain regions as a result of a reorganized neuronal network structure. Whether these types of changes in DMN configuration result from the prior cognitive task remains unclear. Some studies have indicated that the spatial distribution of the DMN appears to persist unchanged across active and resting states, suggesting that the DMN reflects stable properties of brain-network architecture. However, this question cannot be fully answered without knowing how dynamic DMN connectivity changes across different brain states. Additionally, characterization of this dynamic DMN activity is critical for understanding brain functional stability and flexibility.

Indeed, characterizing brain-network topology plays an important role in understanding cognitive function^51^,^52^. Many researchers have explored the relationship between complex topological properties of functional brain networks and cognitive or behavioural measures^27^,^41^,^43^,^47^,^53^,^54^. Moreover, recent studies show that the community structure of the human brain network is associated with cognitive function^47^,^55^. Although many studies having investigated functional connectivity of the DMN during different tasks or at rest, none have similarly assessed DMN topological measures^8^,^13^,^19^,^22^,^36^. Furthermore, from the perspective of a brain network, the dynamic reorganization of DMN functional connectivity in response to an external cognitive task depends on topological properties.

Determining the properties of DMN topology is an important step toward understanding the mechanism of its functional reconfiguration. Here, we used complex network-analysis techniques to investigate the properties of global and nodal DMN topology across different brain states. Specifically, we answered the following questions: (1) Are changes in the topological structure of the DMN associated with different brain states? (2) If so, are these changes modulated by recent cognitive task performance?

## Methods

### Ethics statement and participants

All participants gave their written, informed consent before the experiment was conducted in compliance with an experimental protocol approved by the Ethics Committee of University of Trento, Italy. Experiments were carried out in accordance with the approved guidelines. Fourteen right-handed participants (mean age: 27.4 years, range: 23–35 years) were recruited for the experiment. All participants were healthy and without a history of neurological or psychiatric disorders.

### Experimental design

**Figure 1:**
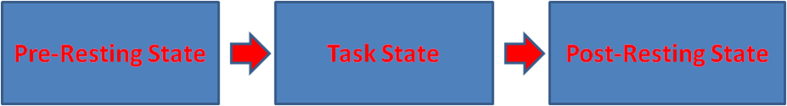
Experimental design. Task design order: pre-resting state, attention task, and post-resting state. For both resting-state conditions, participants were instructed to lie still, relax, and close their eyes. During the cognitive task condition, participants performed a visual attention task.

To explore dynamic DMN functional connectivity across different brain states, we conducted three separate experiments. Each experiment consisted of a 10-min fMRI scan. First, participants underwent a 10-min resting-state scan before an attention task. They were instructed simply to keep their eyes closed and to not think about anything in particular. After the 10-min resting-state scan, participants performed a visual attention task that was administered in blocks. A detailed account of this task has been published elsewhere^5^. Briefly, participants were instructed to respond as quickly as possible to a lateralized visual target with an ipsilateral or a contralateral button press, according to non-verbal visual instructions (a square or diamond, respectively) that they saw at the beginning of each trial within a block. A masked instruction (smaller square or diamond) or neutral shape (star) subliminally preceded each visible instruction. After completing the attention task, participants sat through another 10-min resting-state scan. Figure 1 shows the experimental design.

### MRI data acquisition

Scanning was performed on a 4-T Bruker MedSpec Biospin MRI scanner with an eight-channel phase-array receiver head coil. To make sure the participants fit comfortably inside the scanner during the experiment, we fixed their heads with foam cushions to minimize head movement. The T1-weighted high-resolution anatomical images were acquired using a sagittal magnetization-prepared rapid gradient echo (MP-RAGE) three-dimensional T1-weighted sequence optimized for grey-white matter contrast (repetition time [TR] = 2700 ms, echo time [TE] = 4.18 ms, inversion time [TI] = 1020 ms, flip angle [FA] = 7°, slice number = 176, and matrix size = 224 × 256, 1 × 1 × 1 mm^3^, GRAPPA iPAT = 2). The functional images were acquired using an echo-planar (EPI) sequence corrected for distortion using the PSF method (TR = 2500 ms, TE = 33 ms, FA = 73°, 34 slices, 3 × 3 × 3 mm voxels, field of view [FOV] = 192).

### Data Analysis

#### Functional connectivity preprocessing

The following preprocessing of fMRI data was performed using an analysis based on AFNI (http://afni.nimh.nih.gov/afni/) (Cox 1996) and FSL’s software Library (http://www.fmrib.ox.ac.uk/fsl/). The first four images were removed to avoid T1 equilibration effects. First, the fMRI data were motion-corrected using a linear registration algorithm tool. Then they were spatially smoothed with a Gaussian kernel with a FWHM of 6 mm. Next, we followed the following steps: (1) remove the linear trends; (2) temporally band pass filter (0.009 < f < 0.08) the data and (3) remove several sources of spurious variance from the data through linear regression that incorporated six rigid body motion correction parameters, the white matter signal, and the cerebral spinal fluid (CSF) signal. Recently, whole-brain signal regression has raised challenging interpretive issues. Relatively recent evidence suggests a neuronal origin for the global blood-oxygen-level dependent (BOLD) signal that is typically removed as a nuisance term in resting-state studies. The use of global signal regression is still under debate as a preprocessing step in resting-state fMRI analysis and is not universally recommended^56^,^57^. Here, we did not remove the global whole-brain signal. Each participant’s fMRI data were registered to the MNI152 standard template using FSL’s linear registration algorithm (FLIRT). Additionally, as participant motion can contribute to resting state functional connectivity, we further examined motion across different brain states. Framewise displacement (FD) and temporal derivative of the fMRI time series (DVARS) values were used to identify volumes in the fMRI time data to remove from data analysis. To minimize the motion effects, previous study suggests that each volume movement (FD) exceed the 0.5 mm should be censored^58^. To further compare head motion across pre-task, task, and post-task brain states, we calculated the FD and DVARS across the three brain states. One-way analyses of variance showed that neither FD nor DVARS differed significantly across three brain states (FD: p = 0.1075; DVARS: p = 0.911; Fig. S1).

#### Functional connectivity-mapping analysis

For each participant, seed-based functional connectivity (FC) analysis was conducted by extracting the time series from the PCC region. The seed was constructed using an 8-mm sphere located at (0, −53, 26) in Montreal Neurological Institute (MNI) space, in accordance with a previous study^59^. The correlation coefficients between the PCC-seed time series and those of the entire-brain voxels were calculated. FC maps of the DMN were further derived by Fisher’s r-to-z transformation. For the group-level analysis, a one-sample t-test was conducted to detect brain regions showing significant FC across participants. The group-level FC maps were further corrected for multiple comparisons using FWE-correction with a corrected threshold of *p* < 0.05.

#### DMN region of interest definition and dynamic correlation matrix construction

**Figure 2:**
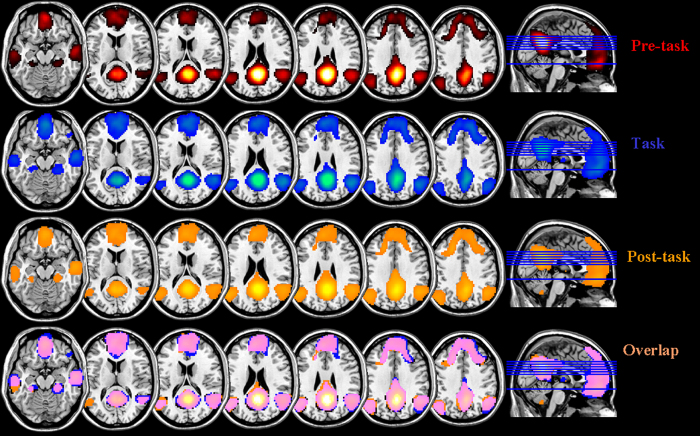
Mapping of DMN functional connectivity in different brain states (corrected *p* < 0.05).

**Figure 3:**
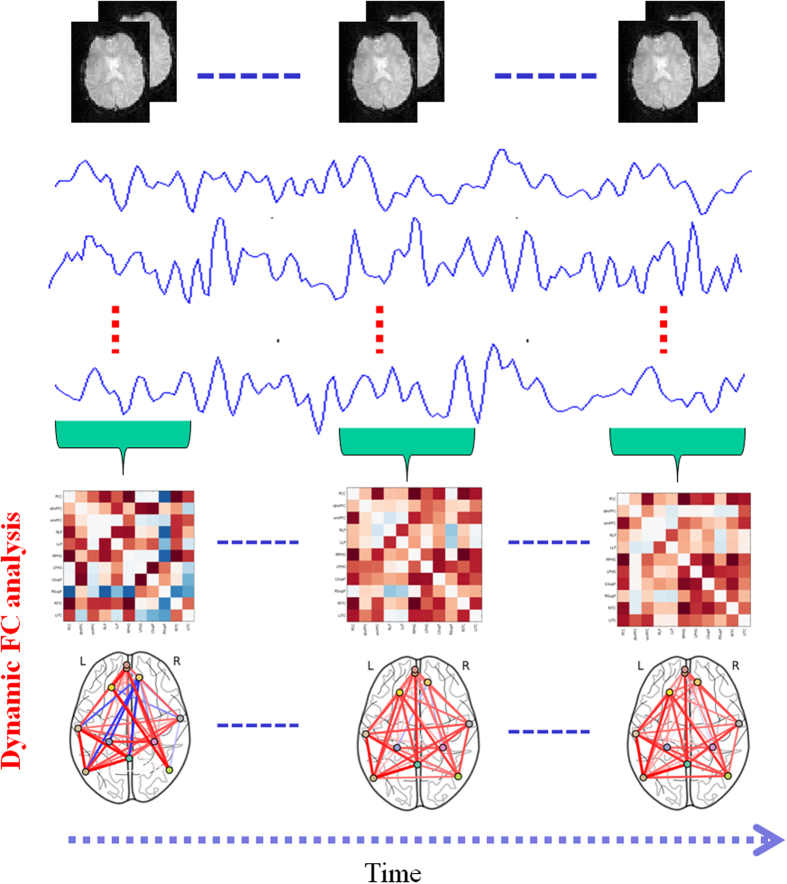
General scheme for the characterization of dynamic DMN, including the construction of dynamic correlation matrices and DMN topology analysis. Topology metrics included nodal degree, clustering coefficient, local efficiency, and global efficiency.

Here, group-based z-score maps of the PCC were corrected for multiple comparisons using FWE-correction for peak voxels. The corrected threshold was set at p < 0.05. The centres of each DMN region of interest (ROI) were determined by considering the coordinates of the z-value local maxima on the correlation map (see Fig. 2). For all DMN ROIs, an 8-mm sphere was created at the peak coordinates of the pre task resting-state FC mapping of the DMN. Thus, the following default network regions were defined in MNI space: posterior cingulate cortex (PCC; MNI coordinate: 0, −53, 26), dorsal medial prefrontal cortex (dmPFC; MNI coordinate: −3, 55, 22), ventral medial prefrontal cortex (vmPFC; MNI coordinate: −3 59, −7), left parahippocampal gyrus (LPHG; MNI coordinate: −24, 33, −27), right parahippocampal gyrus (RPHG; MNI coordinate: 30, −33, −27), left lateral parietal cortex (LLP; MNI coordinate: −52, 69, 26), right lateral parietal cortex (RLP; MNI coordinate: 48, −67, 36), left superior frontal cortex (LSupF; MNI coordinate: −21, 32, 47), right superior frontal cortex (RSupF; MNI coordinate: 12, 44, 48), left inferior temporal cortex (LITC; MNI coordinate: −61, −17, −30), and right inferior temporal cortex (RITC; MNI coordinate: 61, −5, −25). The mean time series for each ROI was then calculated by taking the mean of the voxel time series within each region. Dynamic Pearson’s correlation coefficients were computed between the time series for all pairs of brain regions for each participant based on different sliding windows (60 s, 75 s, and 90 s), and sliding within a step of one TR. Then, 11 × 11 dynamic correlation matrices were generated for all participants. For further statistical analysis, a Fisher’s r-to-z transformation was applied to improve the normality of the correlation coefficients (see Fig. 3).

#### Dynamic Network Analysis

Graph theory is a useful tool for brain-network analysis. To date, most graph measures have only been defined for the simplest case of an unweighted graph, such as by setting all edges with a weight above a certain threshold to binary. However, an unweighted graph approach has several disadvantages. For example, much of the information available in the weights is not used. To better understand the complex brain systems under study, information about the nature and strength of the underlying node interactions should be taken into account to build the weighted network. Thus, we characterized the dynamic changes in the coordinated pattern of DMN networks with a weighted-network analysis. The dynamic correlation matrix for each participant and each sliding window was then obtained based on this analysis. We set the negative functional connectivity to zero. We used this weighted-graph method to calculate the topological metrics of the DMN, and then characterized the dynamic temporal-topological metrics for each sliding window using a weighted complex network analysis that was based on the BCT Matlab toolbox (http://www.brain-connectivity-toolbox.net) (see Fig. 3). The network topology metrics used in this study included nodal degree, clustering coefficient, local efficiency, and global efficiency.

#### Statistical Analysis

We calculated the mean topology metrics (global and local efficiency, clustering coefficients, and degrees of topology metrics) across participants. To further assess the differences in brain-network topological properties across different brain states, the four metrics were statistically evaluated using the Wilcoxon rank sum test. Significant differences in their distributions across different brain states were calculated using the Kolmogorov–Smirnov test. In order to characterize the patterns of dynamic DMN topology across different brain states, we further used the k-means approach to analyse the topology metrics. The K-means approach is one of the simplest and fastest unsupervised learning techniques for clustering, and has been widely used to analyse numerous pattern-recognition problems. For more details regarding the k-means approach, see the supplemental Methods. To assess the relationships between whole-DMN topology metrics within different brain states, we used a robust regression approach that yields valid p-values while minimizing the influences of outliers.

## Results

### Functional connectivity mapping of the DMN across different mental states

Figure 2 shows the z-map for group functional connectivity of the posterior cingulate cortex (PCC) seed across different mental states (FWE-corrected; at a threshold of *p* < 0.05).

### Dynamic DMN nodal network topology

**Figure 4:**
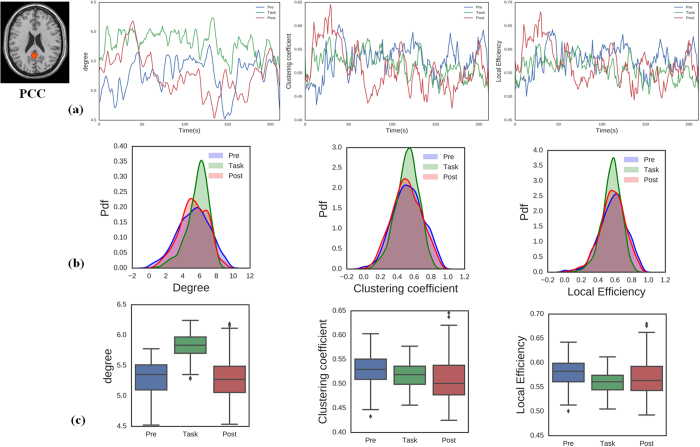
Temporal dynamic topology metrics of the PCC node across different brain states (pre-task resting state, task state, and post-task resting state). (**a**) Properties of PCC topology metrics for nodal degree, clustering coefficient, and local efficiency. (**b**) The probability distribution functions for PCC nodal topology metrics differed significantly across different brain states (two-sample Kolmogorov–Smirnov test, *p* < 0.001). (**c**) Boxplots of the PCC topology metrics indicating that the metrics differ significantly across brain states (Wilcoxon rank sum test, *p* < 0.05).

**Figure 5:**
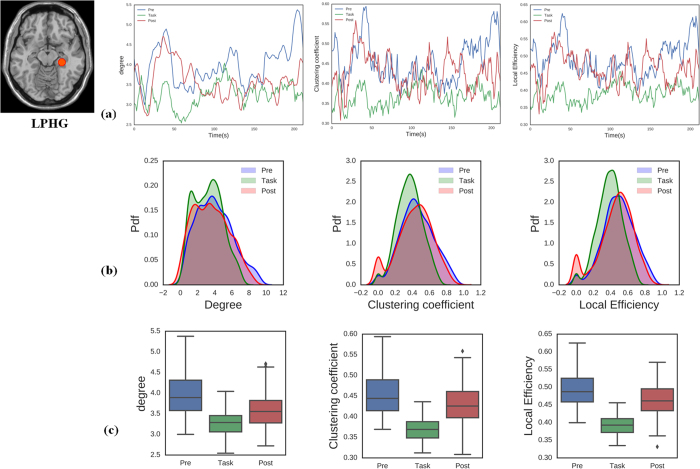
Topology metrics over time for the LPHG node during the pre-task resting state, task state, and post-task resting state. (**a**) Properties of LPHG topology metrics for nodal degree, clustering coefficient, and local efficiency. (**b**) The probability distribution functions for LPHG nodal topology metrics differed significantly across brain states (two-sample Kolmogorov–Smirnov test, *p* < 0.001). (**c**) Boxplots of the LPHG topology metrics indicating that the metrics differ significantly across brain states (Wilcoxon rank sum test, p < 0.05).

To investigate the properties of dynamic DMN nodal topology, we considered three metrics for data analysis (nodal degree, clustering coefficient, and local efficiency). The DMN can be viewed as being composed of sub-networks^60^, including the medial temporal sub-network associated with memory-related nodes (left and right parahippocampal gyri; LPHG and RPHG) and another sub-network associated with the PCC. Here, we primarily focused on analysing these two sub-networks during three different brain states (pre-task resting state network, task, post-task resting state network). Figures 4a and 5a show the average topology of each metric for the PCC and LPHG nodes across the three brain states. We calculated the statistical properties for the temporal evolution of the topology metrics. Figures 4b and 5b show that that the probability distribution functions for the PCC and LPHG topology metrics across all participants were significantly different from each other during the different brain states (*p* < 0.05, Kolmogorov–Smirnov test). Nodal degree, clustering coefficient, and local efficiency were all significantly altered across different brain states (Figs 4c and 5c, Wilcoxon rank sum test, *p* < 0.05). Other metrics of DMN nodal topology show similar patterns (see Supplementary Figs S2–S10). These results indicated that DMN nodal topology can become reorganized as brain states transition back and forth.

**Figure 6:**
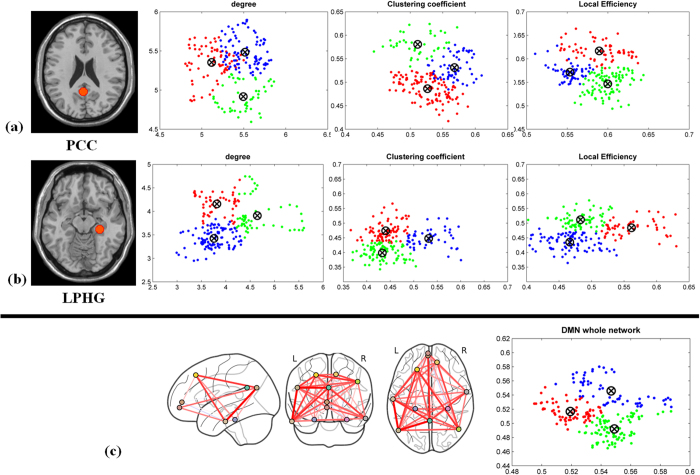
K-means clustering for DMN topology metrics analysis across different brain states. (**a**–**c**) The DMN nodal and global topology metrics formed a separate cluster for each brain state.

To further investigate whether the DMN topology metrics exhibit different patterns as a function of brain state, we analysed them using the k-mean clustering approach. Figure 6a and b show three clusters of topology metrics between different brain states, indicating that DMN nodal topology is dynamically linked to brain state. The other nodal topology metrics show similar patterns (see the Supplementary Fig. S11).

### Dynamic DMN global network topology

**Figure 7:**
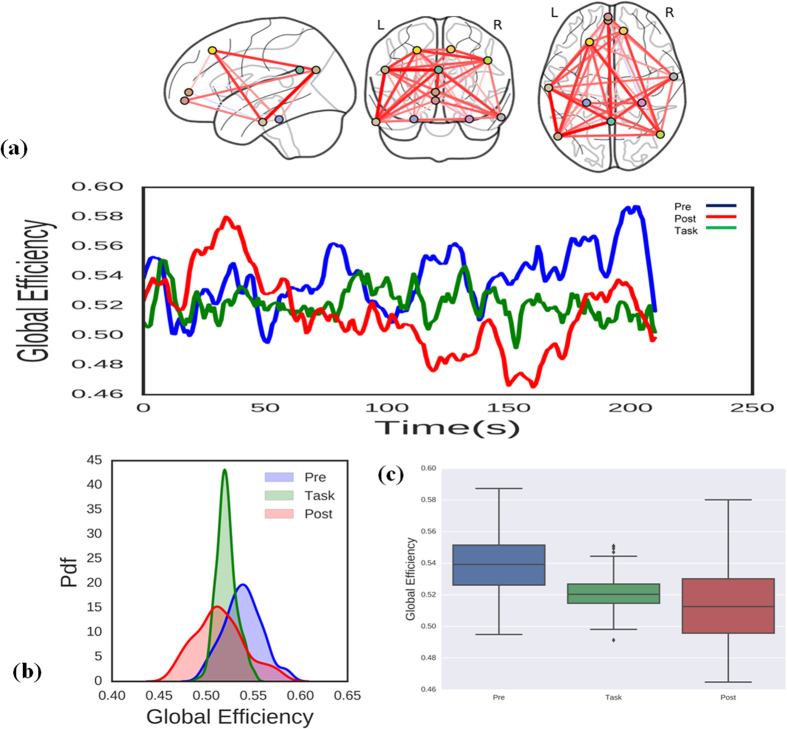
Changes in the DMN global efficiency during the pre-task resting state, task state, and post-task resting state. (**a**) topological properties of DMN global efficiency. (**b**) probability distribution functions for global efficiency. The distribution functions differed significantly (two-sample Kolmogorov–Smirnov test, *p* < 0.001). (**c**) Boxplots of the DMN global efficiency indicating that it differed significantly across brain states (Wilcoxon rank sum test, *p* < 0.05).

We also investigated whether global DMN topology metrics differed depending on brain state. Figure 7a shows the temporal properties of global DMN topology during the pre-task resting state, task state, and post-task resting state. Figure 7b shows that the probability distribution functions for the global topology metrics differed significantly across diverse brain states (*p* < 0.05, Kolmogorov–Smirnov test). Further analysis showed that the task altered DMN global topology (Fig. 7c, Wilcoxon rank sum test, *p* < 0.05), with the DMN global topology metrics for the three brain states forming three separate clusters. These results indicate that the DMN global network topology is also dynamically linked to brain state.

### The relationship between topology structures across different brain states

**Figure 8:**
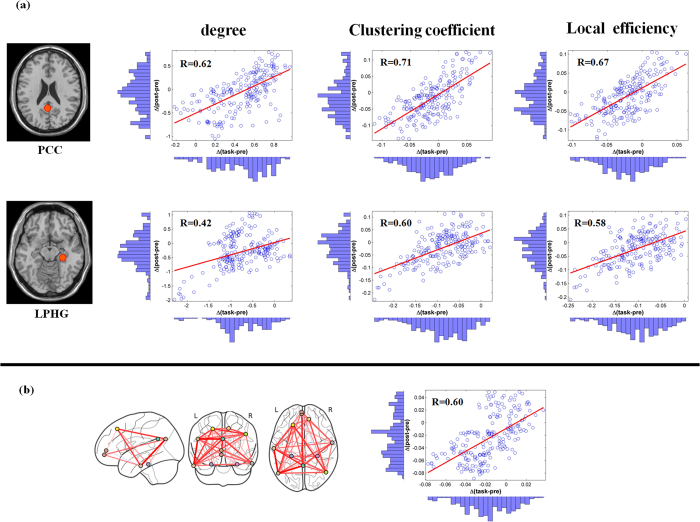
Scatterplots of the association between Δ(task-pre) and Δ(post-pre) in the DMN. (**a**) DMN nodal topology metrics show significant correlation between Δ(task-pre) and Δ(post-pre) in the PCC and LPHG across brain states (*p* < 0.0001). (**b**) DMN global topology metrics show significant correlation between Δ(task-pre) and Δ(post-pre) across brain states (*p* < 0.0001).

To explore the relationship between changes in topology metrics and different brain states, we used a robust regression approach. The change in network topology metrics Δ (task-pre) induced by task performance was associated with the change in network topology metrics Δ (post-pre). Our results show significant positive correlations between Δ (task-pre) and Δ (post-pre) in the PCC, LPHG nodal, and DMN global network topology metrics (see Fig. 8, p < 0.05). The other DMN nodal metrics showed similar results (Supplementary Fig. S12). Thus, the results indicate that task performance significantly modulates DMN topology structure.

### Dynamic DMN topology analysis with different sliding windows

**Figure 9:**
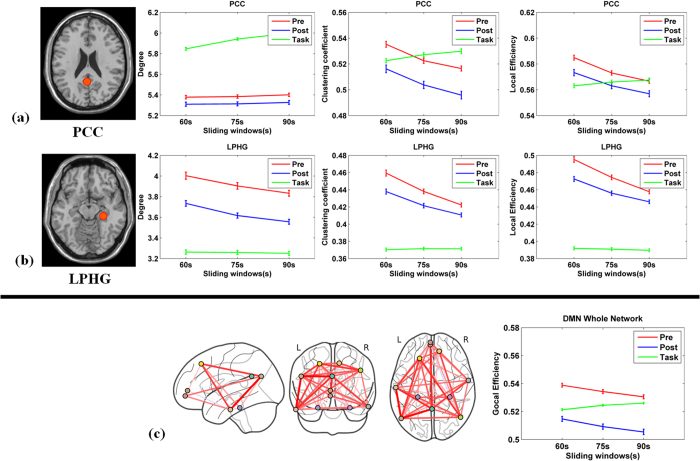
Dynamic DMN network topology metrics for different sliding windows. (**a**–**c**) The PCC, LPHG, and DMN whole-network dynamic topology metrics for each sliding window length (60 s, 75 s, and 90 s). Error bars indicate SEM.

To investigate how the length of the sliding window affects the analysis of DMN topology, we further examined our data using different sliding-window lengths. Previous studies have suggested that longer sliding windows would not be able to adequately characterize dynamic FC properties. Thus, here we focused on shorter sliding-window lengths (60 s, 75 s and 90 s). We consistently observed that DMN nodal and global topology metrics differed significantly depending on task state, regardless of the window length (Fig. 9, Wilcoxon rank sum test, *p* < 0.05). We found similar results for the other DMN regions (Supplementary Fig. S13).

## Discussion

We investigated the structure of DMN topology across different brain states. As we hypothesized, DMN topological structure dynamically changes over time during different brain states. Interestingly, this dynamic organization of nodal and global DMN topology structure can be rebuilt by different brain states. Differences between pre- and post-task DMN topology metrics significantly correlated with differences between pre-task and task DMN topology metrics, thus supporting our second hypothesis and indicating that the topologies of the post-task resting-state brain network are dynamically altered as a function of immediately preceding cognitive experiences. Most importantly, these findings suggest that dynamic changes in network topology within the DMN are related to establishing or reinforcing the most recently acquired novel information.

To date, most stationary FC studies of the DMN have indicated an interplay between the DMN and the dorsal attention network (DAN) during task performance^5^,^24^,^61^, which was associated with searching memory, attention, and spatial learning^62^,^63^. Stationary FC analysis however, may not fully capture the potential variation in correlation patterns over time, and as a result, some of the more subtle information related to network interactions may be lost^33^. Recently, several studies have suggested that the functional connectivity of dynamic brain networks may carry important cognitive and clinically relevant information^33^,^36^,^47^,^64^,^65^,^66^,^67^,^68^. Thus, to better understand the relationship between DMN functional connectivity and brain states over time, the functional connectivity must be investigated across different brain states. The functional connectivity of brain networks during resting and task states depends on time-varying coordination of the cerebral cortex that supports the self-conscious and adaptive processing of the ongoing task^13^,^36^. For example, functional magnetic resonance imaging (fMRI) and magnetoencephalography have revealed a time-varying relationship between the DMN and the DAN that supports human cognitive function^69^. Importantly, optimal behavioural performance is guided by the dynamic changes in the global and local network connectivity structure within the functional networks of the brain^41^.

Furthermore, the DMN topology metrics may index changes in neuronal activity patterns that underlie critical aspects of cognition and clinically relevant information^13^,^33^. For example, dynamic reconfiguration of DMN functional connectivity can track ongoing daydreaming, and changes DMN functional connectivity over time may carry important information associated with behaviour^36^. More importantly, the dynamic properties of network topology are critical for understanding mental disorders^33^. These observations raise the following critical open questions: (1) how can the dynamic-temporal properties of the DMN be characterized? and (2) how does the dynamic DMN configuration change across different brain states? Because the combination of graph analysis and fMRI offers a powerful tool for characterizing complex topological properties of functional brain networks and cognition^52^, determining the dynamic topological properties of the DMN is an important step towards understanding the relationship between DMN functional network organization and cognitive function.

In the present work, we combined a graph-theory approach that incorporated the sliding-windows method to investigate both global and local nodal DMN topology metrics across three brain states (pre-task resting state, task-state and post-task resting state). We observed that the topological structure of DMN local nodal topology metrics (degree, clustering coefficient, and local efficiency) and the DMN global network efficiency metric both dynamically change over time. These results are consistent with prior studies^33^. The fact that interaction within DMN regions was dominated by short intermittent events rather than being the result of a continuously sustained process has important implications for understanding how DMN organization adapts and reconfigures itself in response to an external task stimulus or to a change in cognitive state^36^. The dynamic topological structure of the DMN that we observed could represent a temporal signature of a brain microstate that supports internal and on-going different brain-state cognitive processes^47^,^70^. These results suggest that DMN dynamic properties could be linked to variations in brain-information processing across multiple time scales. Specifically, characterizing the dynamic and adaptive reconfiguration of the DMN may provide greater understanding of both the fundamental properties of normative brain cognition and the pathophysiology of mental illnesses.

The DMN is known to be most active during the resting state, with deactivation occurring during task performance^2^. The DMN shows a similar spatial architecture across rest and a variety of tasks^71^. However, whether its temporal topological pattern change as a function of brain states has been unclear. Here, our results show that metrics for DMN nodal and global network topology are dynamic and significantly differ across different brain states.

Previous studies have demonstrated that the dynamic interactions between DMN, central executive network (CEN) and salience network (SN) play an important role in the shift between resting and focusing attention, which involves the re-allocation of resources within the brain for supporting stimulus-related cognitive processing^2^,^5^,^21^,^63^,^72^,^73^,^74^. The organization of brain network recruitment is thought to occur in response to an external cognitive task. From the perspective of DMN deactivation, studies have shown a decrease in the activity of the DMN during performance of an external task. Additionally, research has found evidence of increased connectivity within the DMN during the resting state compared to the task state. Indeed, a number of studies have proved that the DMN exhibits a tendency to co-modulate during the resting state^46^. Based on this evidence, we expected that DMN topology during task performance would shift to support the ongoing task. In fact, our results show that PCC-, LPHG- and DMN-related nodal topology metrics are dynamic and change during the task state. At the level of global topology, we found that global efficiency decreased during the task state. Global efficiency has been shown to measure the speed of information transfer in the whole network^75^. One possible explanation is the suppression of DMN activity during task performance, which induces a decrease in global efficiency within the DMN. The dynamic configuration of local and global DMN topology metrics plays an important role in supporting cognitive function. A series of recent studies have suggested that dynamic changes in brain network topology are associated with task performance^27^,^39^,^47^,^69^. Our results support the idea that both local and global topology reorganization, in concert, assist in the shift between resting state and task state neuronal processing.

Our findings raise the issue of how a behavioural task can modulate subsequent post-task resting-state DMN topology. Here, we found that post-task resting-state DMN topology metrics exhibited significantly different topological patterns than the task state. The DMN nodal and global network topology metrics significantly changed during the post-task resting state. K-means clustering results indicate that the nodal and global network topology metrics exhibit different patterns during different brain states. Furthermore, switching from the task state to the resting state generated a topological pattern that was opposite what was generates when switching from the resting state to the task state. Previous studies have suggested that the DMN might recover to its pre-task state during the subsequent resting state^76^. Somewhat in contrast, we found that in addition to recovery of the pre-task state, significant differences in local and global DMN network topology metrics between the pre- and post-task resting states. A possible explanation for our finding is that the DMN has been modulated by the prior cognitive task, which would shape the DMN topology properties and lead to the development of a new DMN organization. Substantial change in DMN activity has been found to occur during the transitions from task states to the resting state^68^,^77^,^78^. Moreover, recent studies have shown that DMN activity could be differentially modulated even in the absence of an external task^15^,^79^,^80^. Taken together, our results demonstrate that the patterns of DMN topology vary across different brain states. The characterization of these dynamic DMN topological patterns might thus enable us to trace or distinguish different brain states.

**S**tudies have shown that the post-task resting-state network might be shaped by the performance during a prior task or through training^44^,^45^,^49^. Here, we explored the relationship between post-task resting-state, pre-task resting-state, and task state nodal topology metrics. We found that post-task resting-state DMN nodal and global topology metrics were associated with task-state modulation. In particular, we found that differences between pre-task and post-task PCC and LPHG nodal topology and DMN whole-network metrics significantly correlated with differences between pre-task and task DMN topology metrics. These results suggest that the post-task resting-state DMN nodal and global topologies are related to prior network co-activation. A possible explanation is that prior learning or external task demand changes the DMN sub-network interactions within the DMN, which can lead to reshaping of the new DMN topology properties to fit the new cognitive behaviour. Recently, studies have suggested that post-task resting-state brain network reorganization is dependent on a prior cognitive task, which may be compatible with a long-term memory system^81^. Thus, learning, or experience-dependent alteration, has been observed in spontaneous brain activity following cognitive task performance, and brain activity related to the alterations are associated with the preceding task demands^45^,^77^. Interestingly, Peigneux *et al*. found that offline regional activity was modulated by a recent learning task^82^. The post-training activity significantly correlated with prior task performance^81^. Additionally, clinical studies show that reshaping of the DMN topology is also associated with neuropsychiatric diseases such as schizophrenia, Alzheimer’s disease and alcoholism^18^,^53^,^83^. Thus, temporally coherent dynamic change in the global brain network is dependent on the prior task type. Moreover, the whole-network topology changes smoothly over a range of temporal scales. Taken together with our findings, this shows that DMN global network topology metrics may be modulated under different brain states.

In conclusion, the topology of the DMN changes over time as individuals’ transition between resting and task states. The nodal and global topological structure is reshaped in different brain states and shows different patterns. Furthermore, the topology metrics are dynamically altered as a function of cognitive experiences, and the modulated networks are assembled in the subsequent resting state. These findings suggest that understanding the dynamic topology of the DMN can provide a new way to better characterize DMN function.

## Additional Information

**How to cite this article:** Lin, P. *et al*. Dynamic Default Mode Network across Different Brain States. *Sci. Rep.* **7**, 46088; doi: 10.1038/srep46088 (2017).

**Publisher's note:** Springer Nature remains neutral with regard to jurisdictional claims in published maps and institutional affiliations.

## Supplementary Material

Supplemental Materials
