# Supplementary material for: Dynamic Default Mode Network across Different Brain States
Source: Sci Rep. 2017 Apr 6;7:46088. doi: 10.1038/srep46088 (PMC5382672; doi:10.1038/srep46088)
Supplement: Supplemental Materials [file srep46088-s1.doc]

**Supplemental Material**

# Dynamic Default Mode Network across Different Brain States

Pan Lin^1,3,4,5†^,Yong Yang^2^, Junfeng Gao^1†^, Nicola De Pisapia^4^, Sheng Ge^5*^, Xiang Wang^6^, Chun S. Zuo^7^, James Jonathan Levitt^8,9^ , Chen Niu^10*^

# The K-means algorithm

The K-means algorithm is one of the simplest and the fastest unsupervised learning techniques for clustering, and has been widely used for numerous pattern recognition problems. The algorithm is an iterative clustering method that produces an optimal *K* partition $C_{1}, C_{2},\cdots, C_{K}$, which minimizes the following function (1):

$$J=\sum_{i=1}^{N} \sum_{k=1}^{K} \mu\left(x_{i}\in C_{k}\right) d^{2}\left(x_{i},v_{k}\right)$$

where $\left\{x_{1},x_{2},\cdot\cdot,x_{N}\right\}\subseteq R^{p}$, $N$ is the number of data items, $K$ is the number of clusters with $2\leq K<N$, equals to 1 if it is true and 0 otherwise, $v_{k}$ is the prototype of the centre of cluster $k$, $d^{2}\left(x_{i},v_{k}\right)$ is a distance measure between object $x_{i}$ and cluster centre$v_{k}$.

Reference:

Tzortzis GF and Likas AC (2009) The global kernel k-means algorithm for clustering in feature space. *IEEE Transactions on Neural Networks*. 20(7);1181-1194.


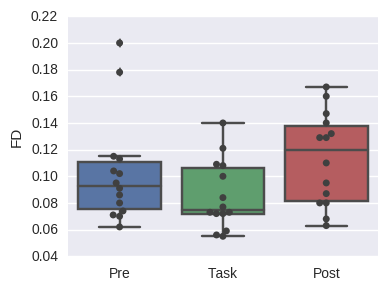

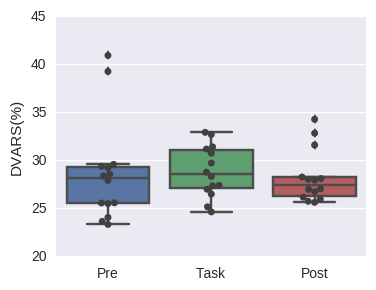


1. (B)

Fig.S1 Head motion parameters across different brain states. (A) Mean FD <0.5mm and FD did not differ across brain states (one-way ANOVA, *p* = 0.1075). (B) DVARS did not differ across brain states (one-way ANOVA, *p* = 0.911).

#
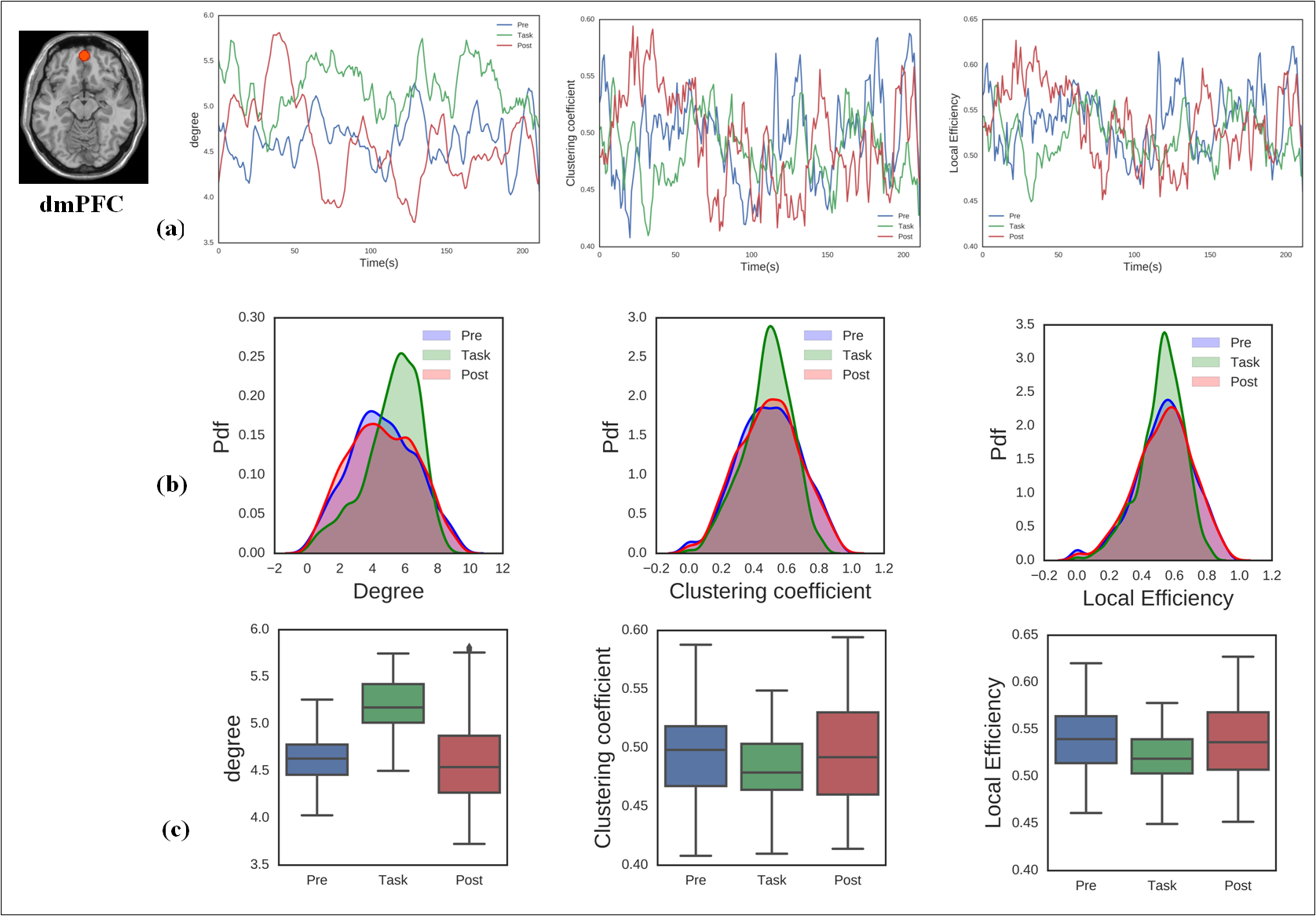


Fig.S2 Topology metrics for the dmPFC node over time across brain states (pre-task resting state, task state, and post-task resting state). (a) Dynamic dmPFC topology metrics including degree, clustering coefficient, and local efficiency. (b). The probability distribution functions of dmPFC nodal topology metrics across different brain states. All comparisons of the probability distribution functions showed significant differences (two-sample Kolmogorov–Smirnov test, *p* < 0.001). (c) Boxplots of the dmPFC topology metrics for different brain states. The degree differed significantly across brain states (Wilcoxon rank sum test, *p* < 0.05).

#
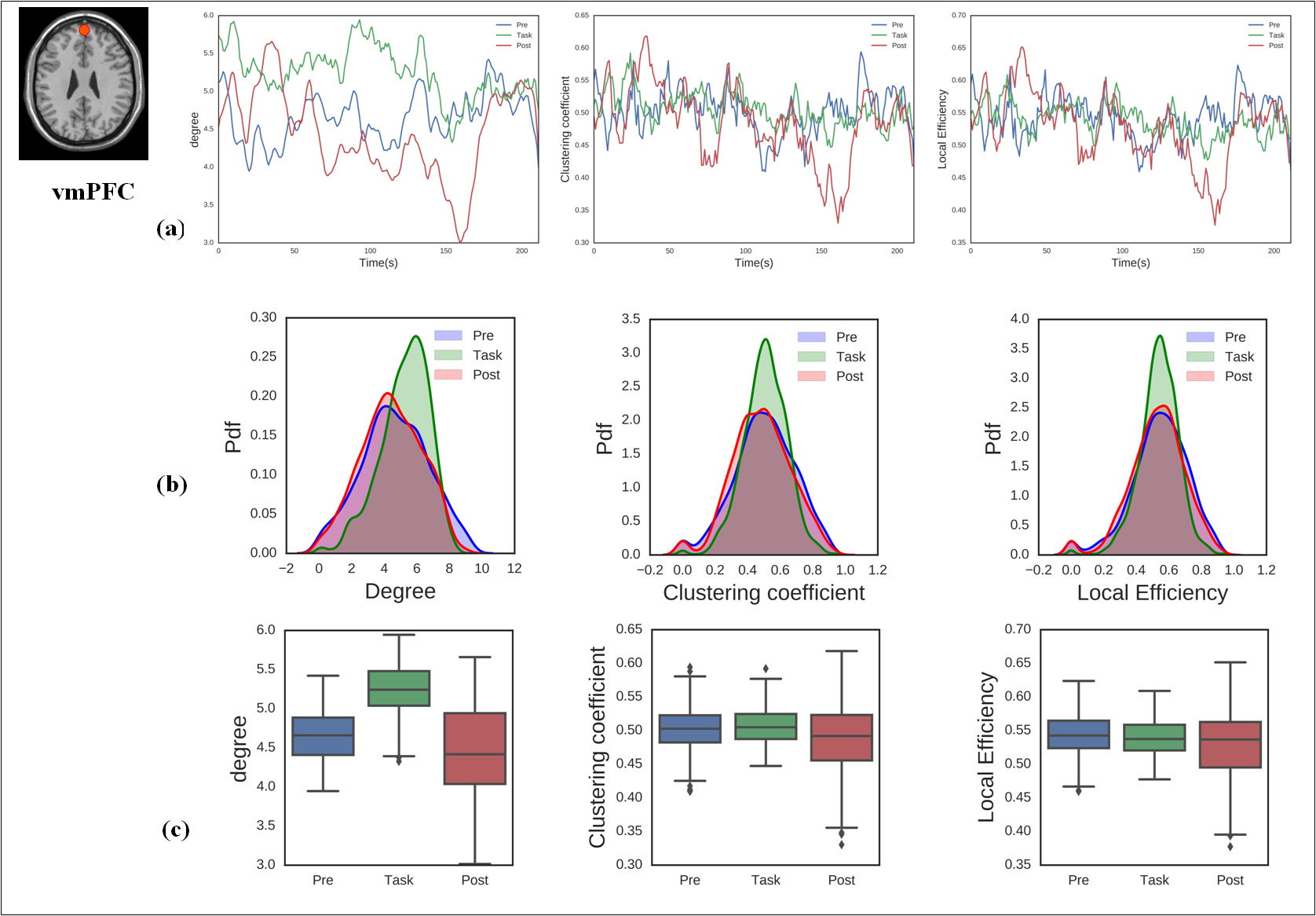


Fig.S3 Topology metrics for the vmPFC node across brain states (pre-task resting state, task state, and post-task resting state). (a) Dynamic vmPFC topology metrics, including degree, clustering coefficient, and local efficiency. (b). The probability distribution functions of vmPFC nodal topology metrics s across brain states. All comparisons of the probability distribution functions showed significant differences (two-sample Kolmogorov–Smirnov test, *p* < 0.001). (c) Boxplots of the vmPFC topology metrics for different brain states. The degree and local efficiency differed significantly across brain states (Wilcoxon rank sum test, *p* < 0.05).

#
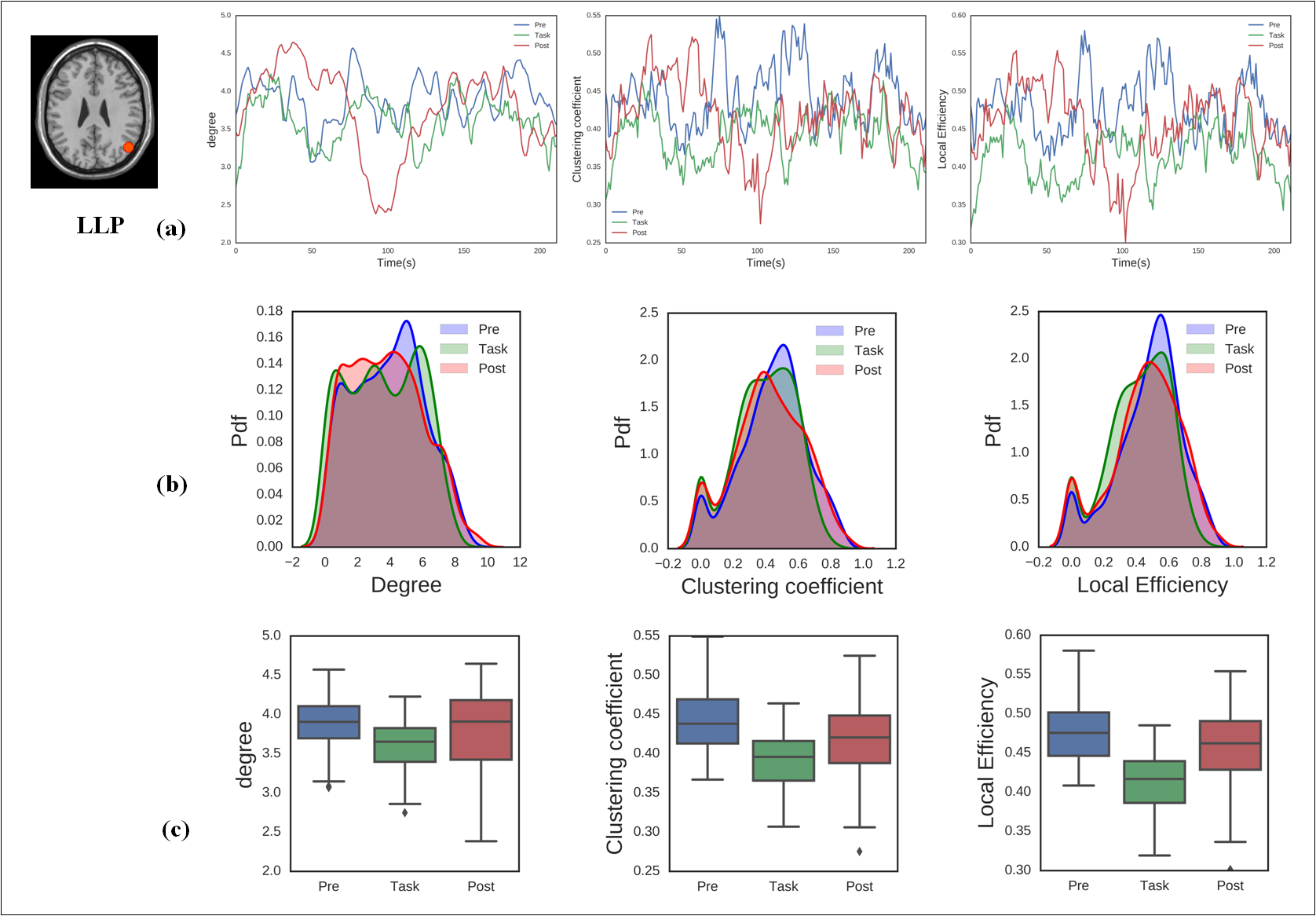


Fig.S4 Topology metrics for the LLP node across brain states (pre-task resting state, task state, and post-task resting state). (a) Dynamic LLP topology metrics, including degree, clustering coefficient, and local efficiency. (b). The probability distribution functions of LLP nodal topology metrics across brain states. All comparisons of the probability distribution functions showed significant differences (two-sample Kolmogorov–Smirnov test, *p* < 0.001). (c) Boxplots of the LLP topology metrics (degree, clustering coefficient, and local efficiency) indicate that they differed significantly across brain states (Wilcoxon rank sum test, *p* < 0.05).

#
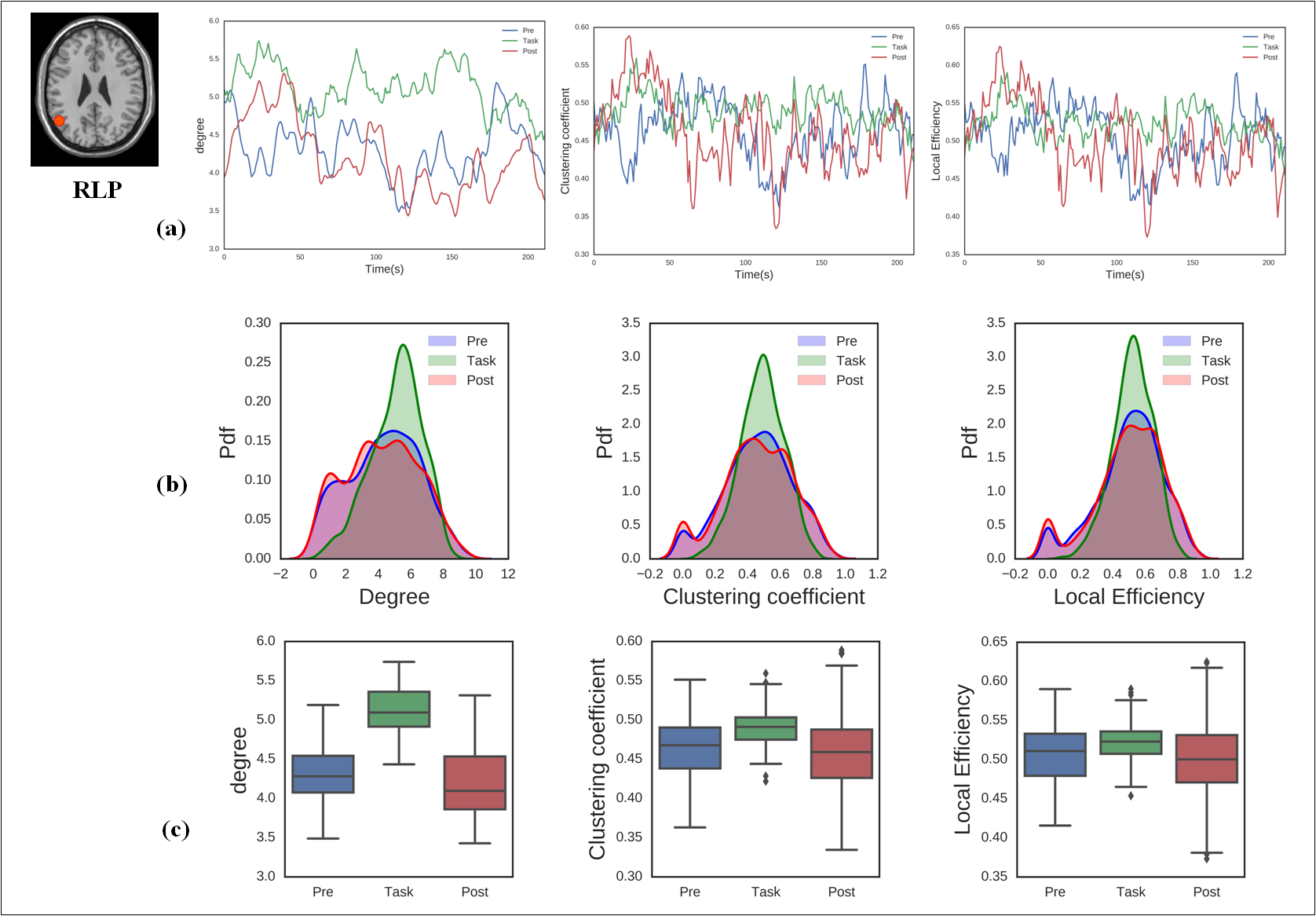


Fig.S5 Topology metrics of the RLP node across brain states (pre-task resting state, task state, and post-task resting state). (a) Dynamic RLP topology metrics, including degree, clustering coefficient, and local efficiency. (b). The probability distribution functions of RLP nodal topology metrics across brain states. All comparisons of the probability distribution functions showed significant differences (two-sample Kolmogorov–Smirnov test, *p* < 0.001). (c) Boxplots of the RLP topology metrics for degree and local efficiency indicate that they differed significantly across brain states (Wilcoxon rank sum test, *p* < 0.05).

#
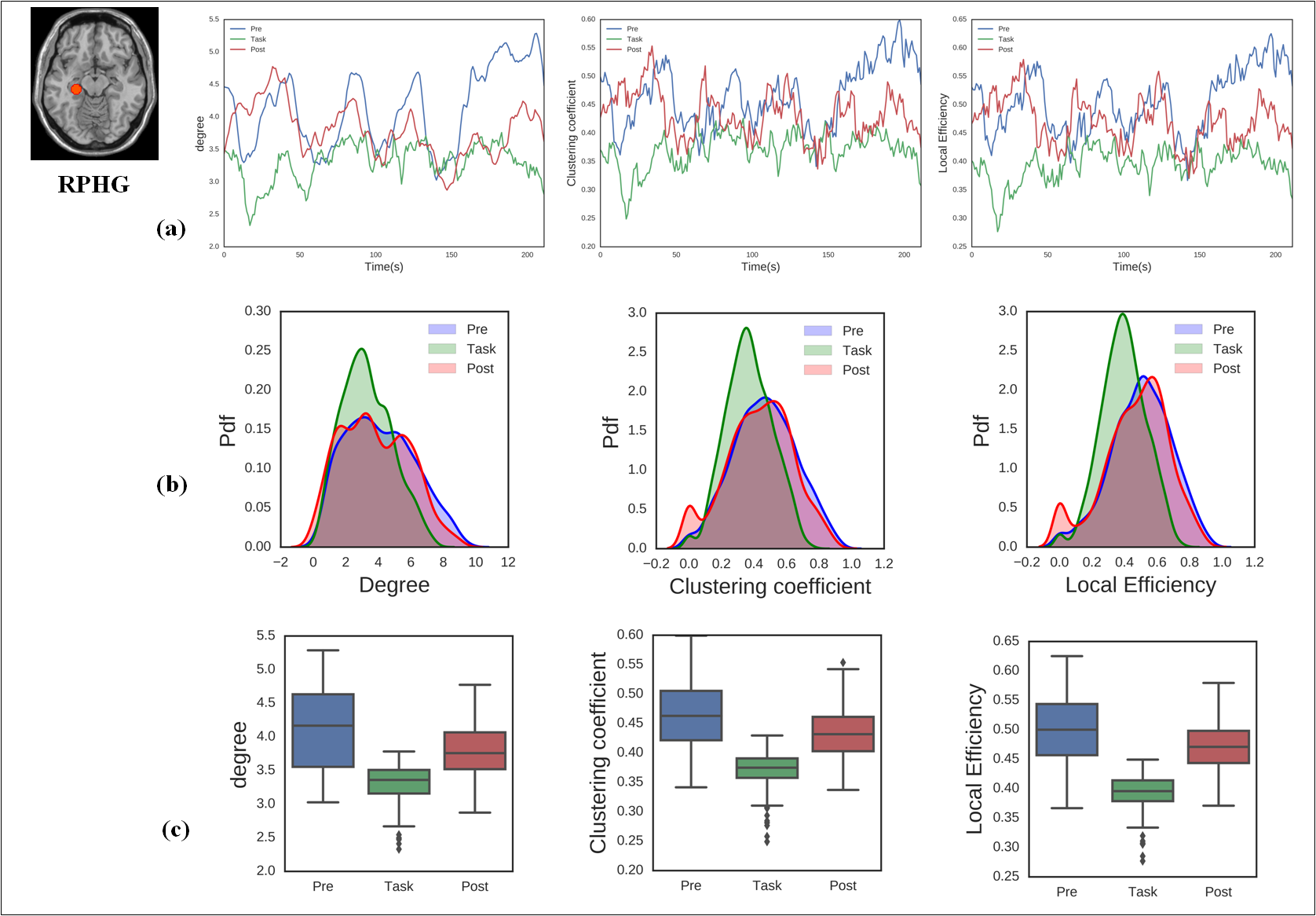


Fig.S6 Topology metrics of the RPHG node across brain states (pre-task resting state, task state, and post-task resting state). (a) Dynamic RPHG topology metrics, including degree, clustering coefficient, and local efficiency. (b). The probability distribution functions of RPHG nodal topology metrics across brain states. All comparisons of the probability distribution functions showed significant differences (two-sample Kolmogorov–Smirnov test, *p* < 0.001). (c) Boxplots of the RPHG topology metrics for different brain states The nodal degree, clustering coefficient, and local efficiency differed significantly across brain states (Wilcoxon rank sum test, *p* < 0.05).

#
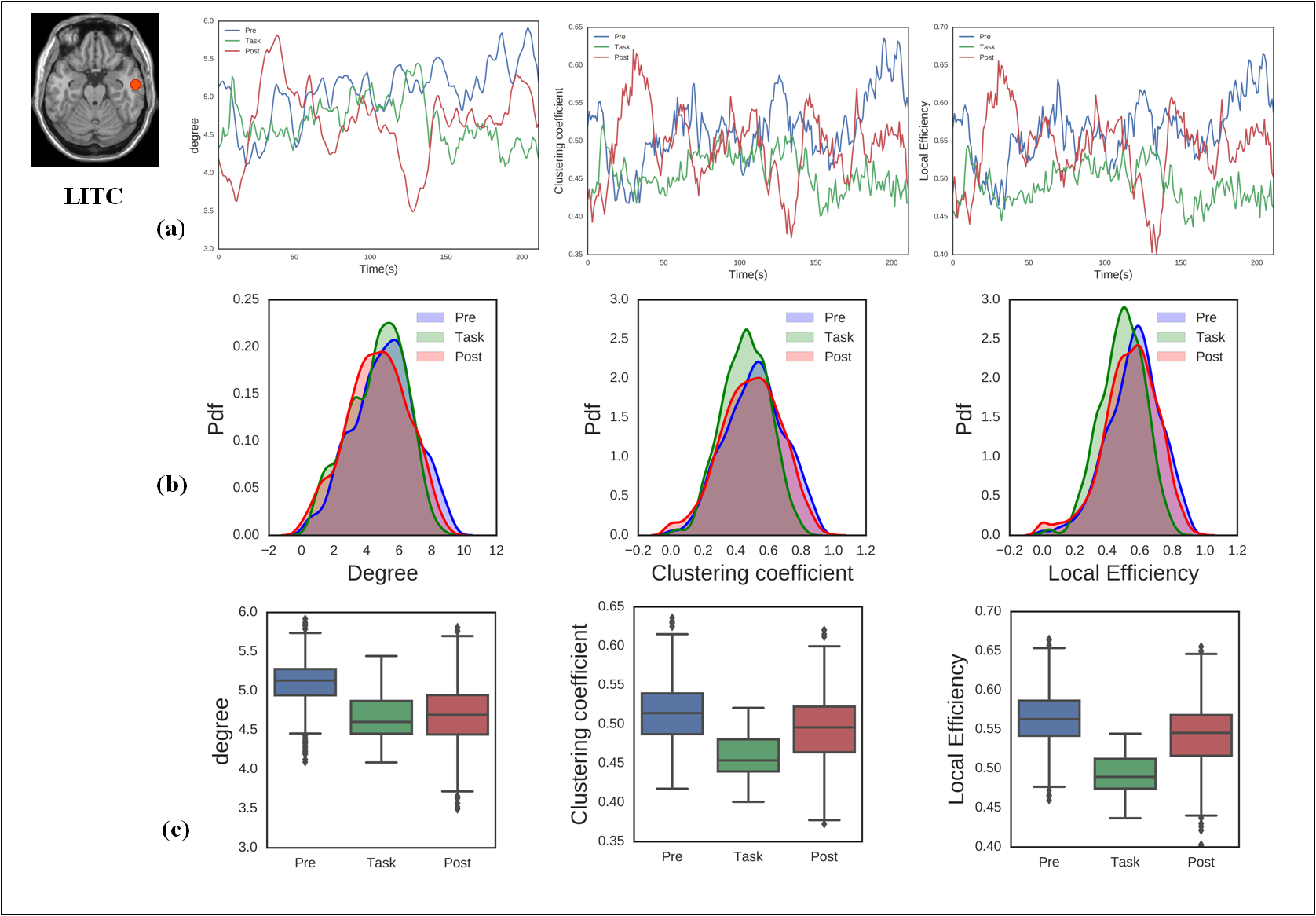


Fig.S7 Topology metrics of the LITC node across brain states (pre-task resting state, task state, and post-task resting state). (a) Dynamic LITC topology metrics, including degree, clustering coefficient, and local efficiency. (b). The probability distribution functions of LITC nodal topology metrics across brain states. All comparisons of the probability distribution functions showed significant differences (two-sample Kolmogorov–Smirnov test, *p* < 0.001). (c) Boxplots of the LITC topology metrics for different brain states. The clustering coefficient and the local efficiency indicate that they differed significantly across brain states (Wilcoxon rank sum test, *p* < 0.05).

#
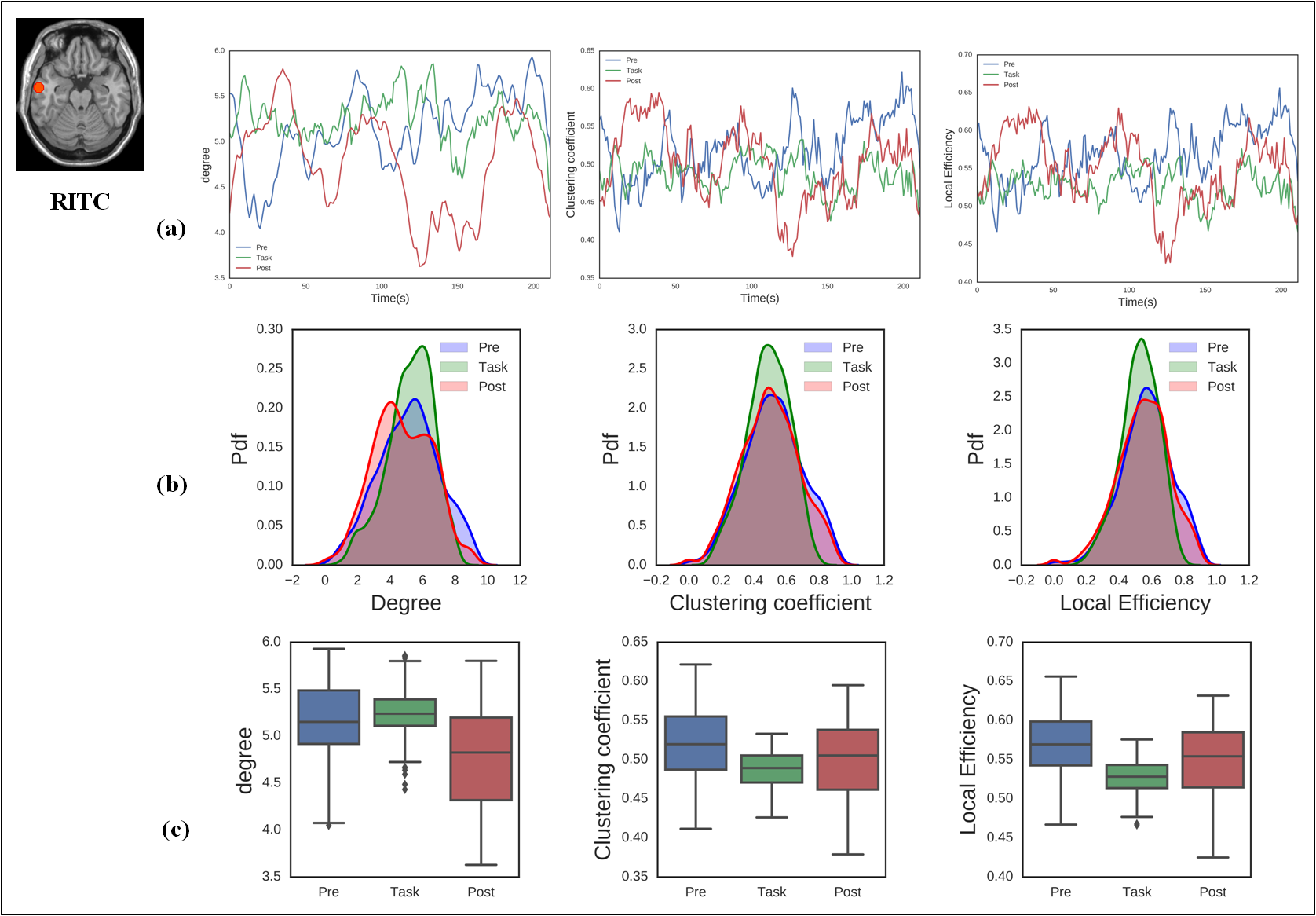


Fig.S8 Topology metrics of the RITC node across brain states (pre-task resting state, task state, and post-task resting state). (a) Dynamic RITC topology metrics including degree, clustering coefficient, and local efficiency. (b). The probability distribution functions of dynamic RITC nodal topology metrics across brain states. All comparisons of the probability distribution functions showed significant differences (two-sample Kolmogorov–Smirnov test, *p* < 0.001). (c) Boxplots of the RITC topology metrics for different brain states. Degree and local efficiency differed significantly across brain states (Wilcoxon rank sum test, *p* < 0.05).

#
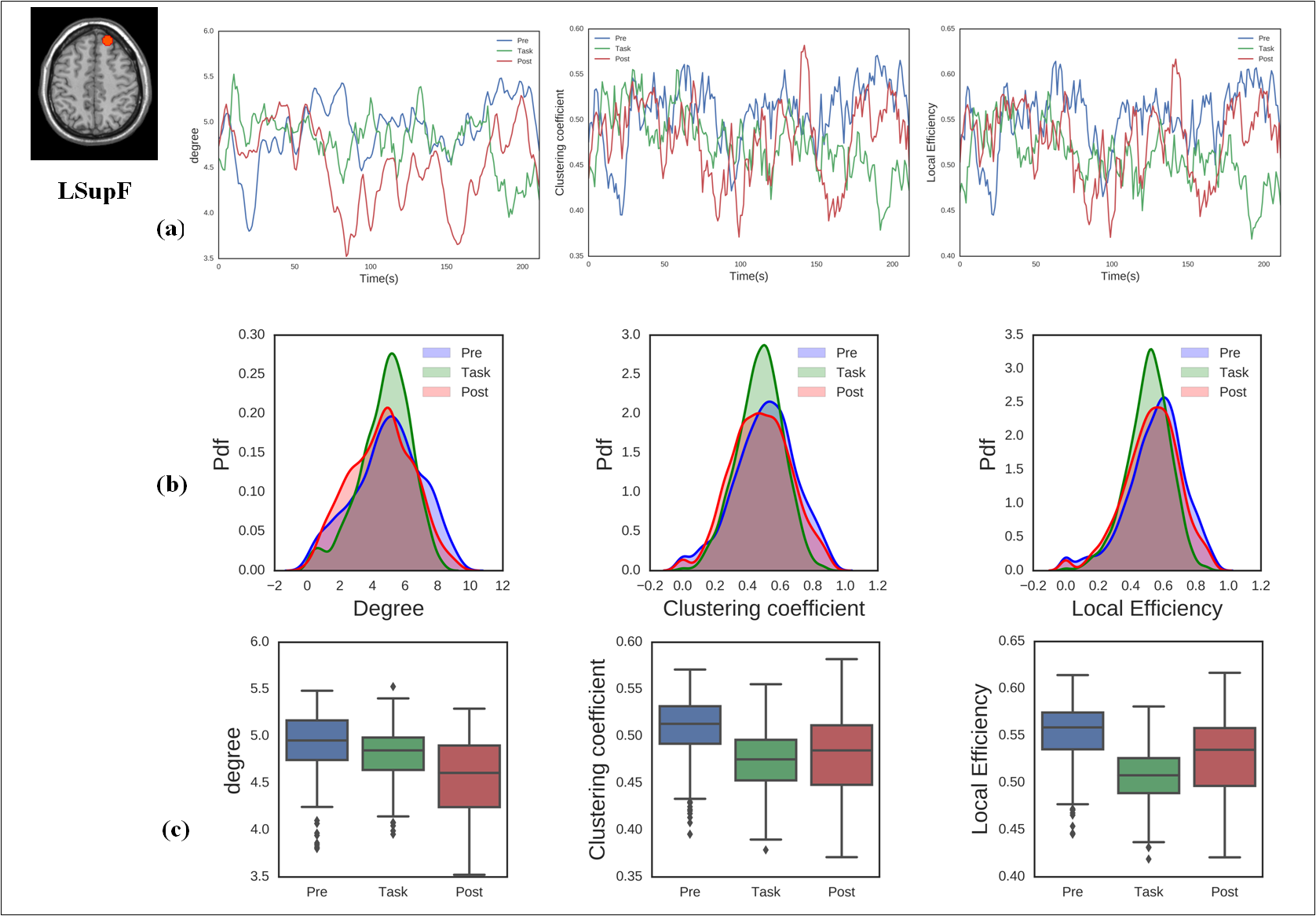


Fig.S9 Topology metrics of the LSupF node across brain states (pre-task resting state, task state, and post-task resting state). (a) Dynamic LSupF topology metrics, including degree, clustering coefficient and local efficiency. (b). The probability distribution functions of LSupF nodal topology metrics across brain states. All comparisons of the probability distribution functions showed significant differences (two-sample Kolmogorov–Smirnov test, p<0.001). (c) Boxplots of the LSupF topology metrics for different brain states. Degree, and local efficiency differed significantly across brain states (Wilcoxon rank sum test, *p* < 0.05).

#
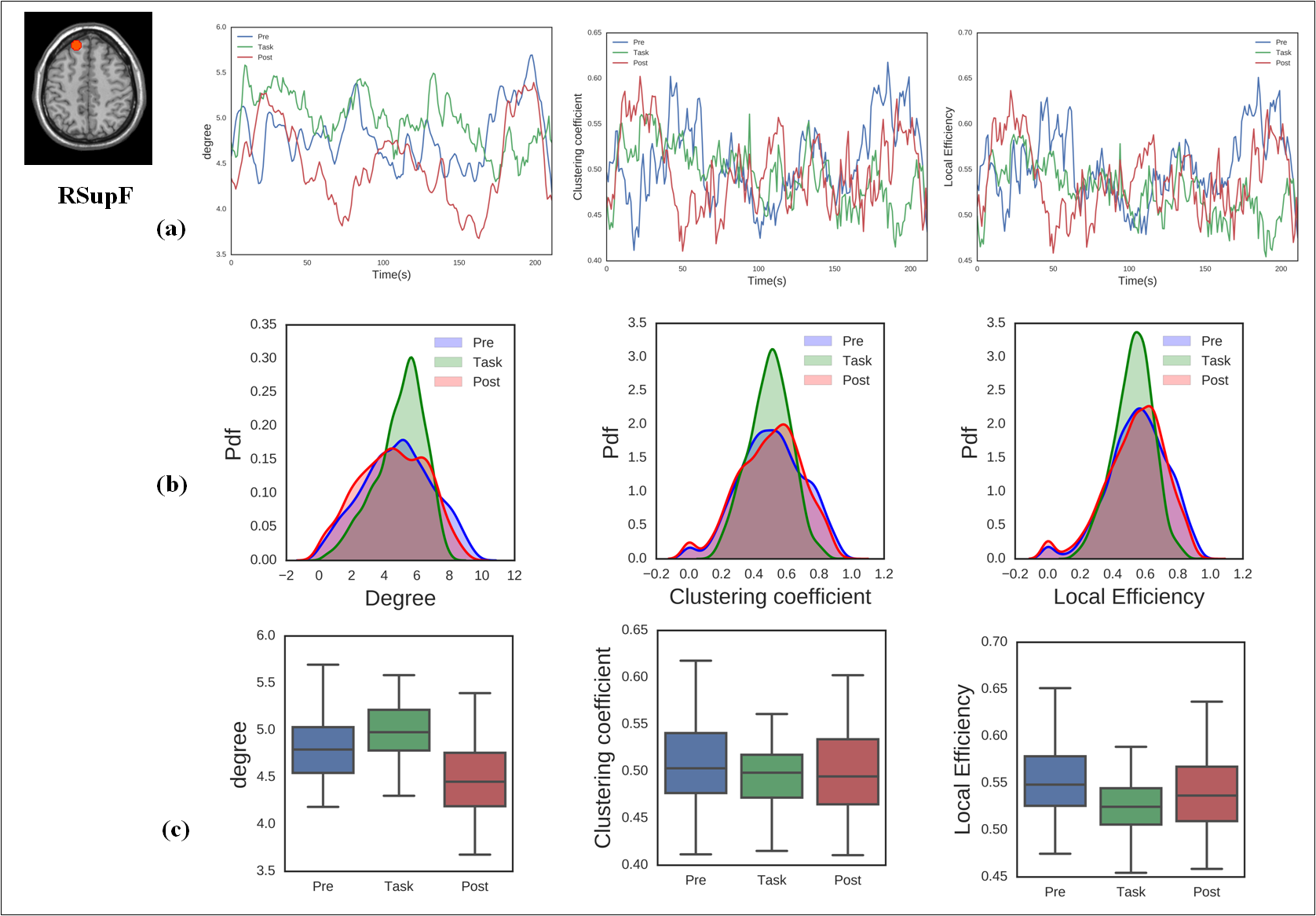


Fig.S10 Topology metrics of the RSupF node across brain states (pre-task resting state, task state, and post-task resting state). (a) Dynamic RSupF topology metrics, including degree, clustering coefficient, and local efficiency. (b). The probability distribution functions of RSupF nodal topology metrics across brain states. All comparisons of the probability distribution functions showed significant differences (two-sample Kolmogorov–Smirnov test, *p* < 0.001). (c) Boxplots of the RSupF topology metrics for different brain states. The nodal degree and local efficiency differed significantly across brain states (Wilcoxon rank sum test, *p* < 0.05).

#
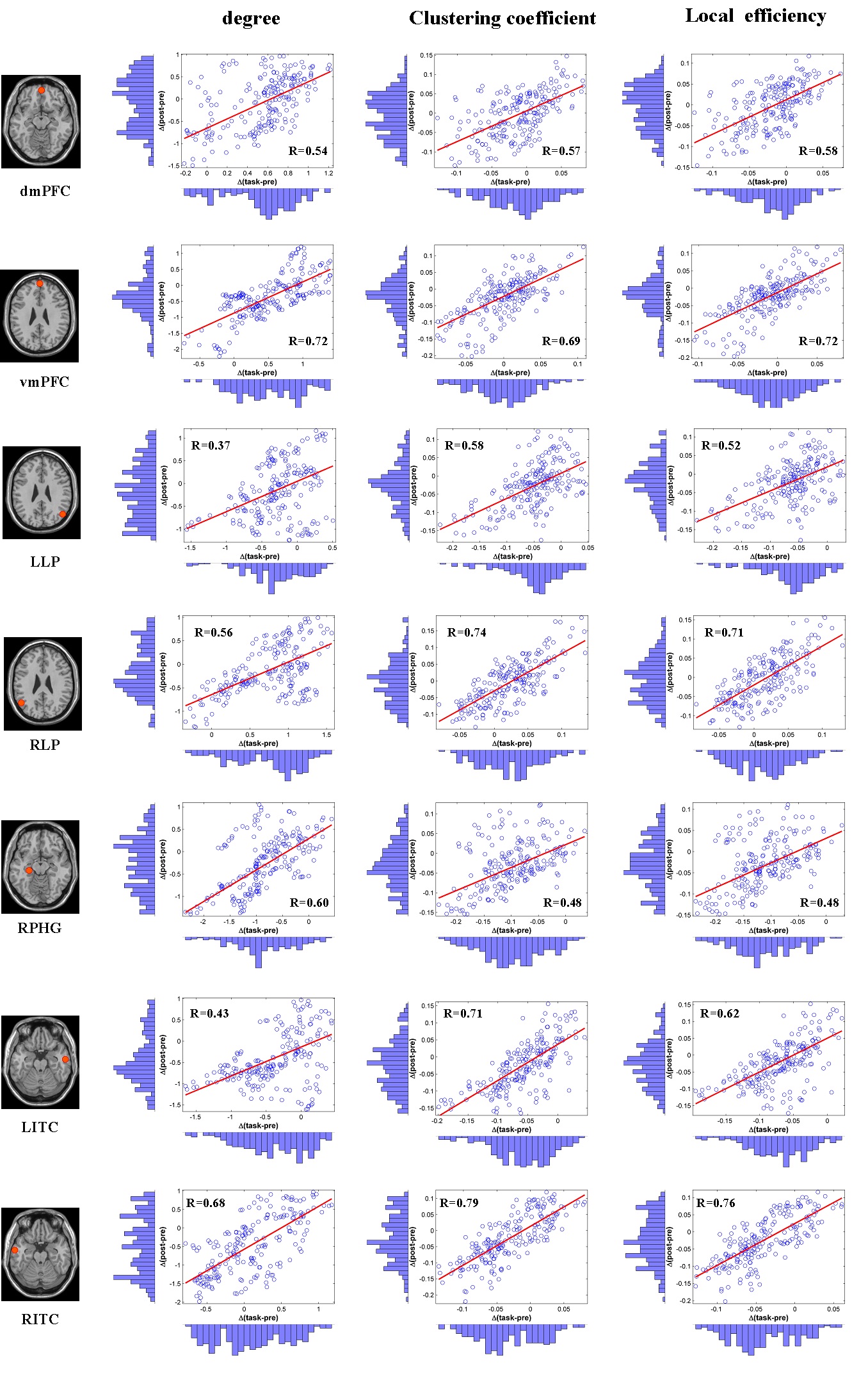


Fig.S11 Scatterplots of the association between Δ(task-pre) and Δ(post-pre) in the DMN. DMN nodal topology metrics show a significant correlation between Δ(task-pre) and Δ(post-pre) in DMN nodes across brain states (*p* < 0.0001).

#
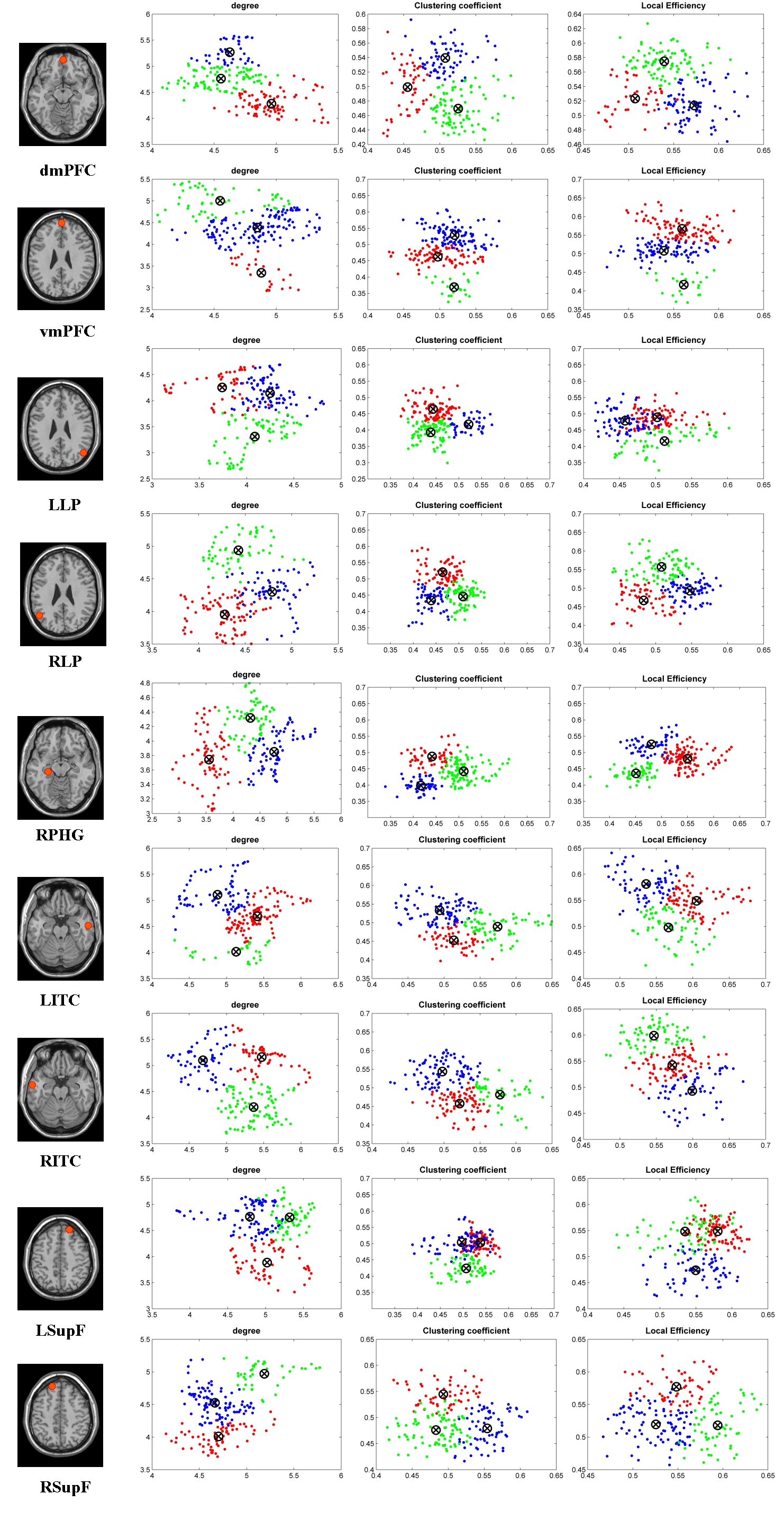


Fig.S12 K-means clustering for analysis of DMN topology metrics across brain states. (a)-(c) The metrics for each state were classified into three separate clusters

#
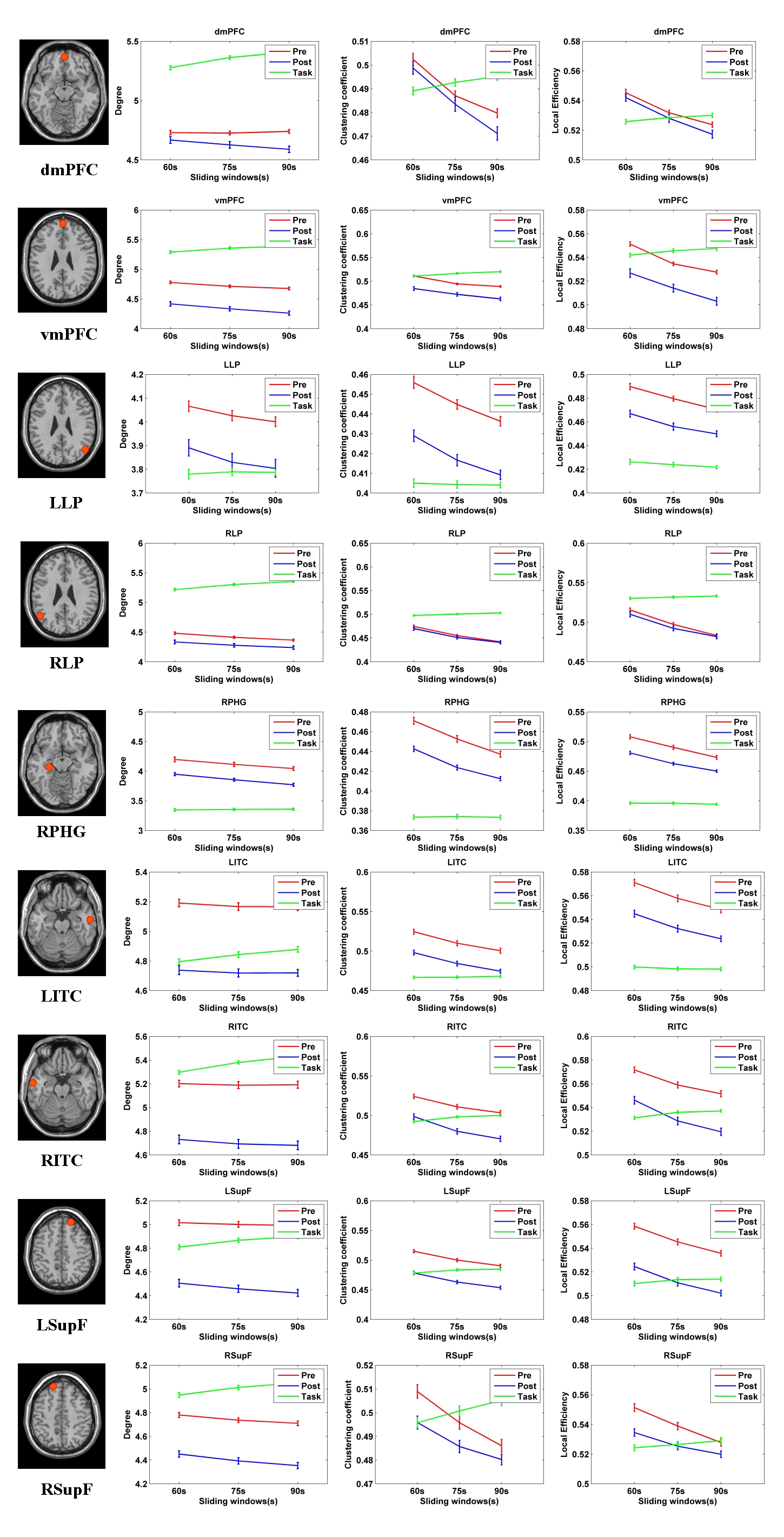


# Fig.S13 Dynamic DMN network-topology metrics for different sliding windows. The plots show the metrics for each sliding window length (60 s, 75 s, and 90 s).
